# Insomnia symptoms among individuals with osteoarthritis and symptoms indicative of osteoarthritis: A population-based cross-sectional study using the CLSA

**DOI:** 10.1371/journal.pone.0322361

**Published:** 2025-05-12

**Authors:** Melissa Cavallo, Elizabeth M. Badley, Anthony V. Perruccio

**Affiliations:** 1 Arthritis Community Research and Epidemiology Unit and Schroeder Arthritis Institute, Krembil Research Institute, University Health Network, Toronto, Ontario, Canada; 2 Dalla Lana School of Public Health, University of Toronto, Toronto, Ontario, Canada; 3 Department of Surgery, Faculty of Medicine, University of Toronto, Toronto, Ontario, Canada; University of Rijeka Faculty of Medicine: Sveuciliste u Rijeci Medicinski fakultet, CROATIA

## Abstract

**Objective:**

To compare the prevalence and examine the likelihood of insomnia symptoms in middle- and older-aged adults with an osteoarthritis (OA) diagnosis and with joint symptoms indicative of OA but without a diagnosis, in a population-based sample.

**Methods:**

Data are from the Canadian Longitudinal Study on Aging (participants aged >45). Individuals reported on doctor-diagnosed OA (hand, hip, or knee) and joint symptoms typical of OA, irrespective of OA diagnosis. A three-level ‘OA-status’ variable was derived: diagnosed OA; joint symptoms-no OA; no symptoms-no OA (controls). Participants responded to sleep-related questions and were categorized as experiencing insomnia symptoms (yes/no). Logistic regression analysis examined the association between insomnia symptoms and OA status, adjusting for a number of covariates.

**Results:**

Of 21,422 respondents, OA was reported by 29.1% (mean age 68.2) and 17.2% reported joint symptoms-no OA (mean age 63.9). One third of those with diagnosed OA and with joint symptoms-no OA reported insomnia symptoms compared to a quarter of controls. Those with OA and with joint symptoms-no OA were similarly more likely to report insomnia symptoms than controls (odds ratio (OR) 1.25, 95% CI 1.17–1.35 and OR 1.32, 95% CI 1.21–1.43). Also significantly associated with insomnia symptoms were female sex, current smoker, lower activity level, multiple chronic conditions and depressive symptoms. Odds ratio magnitudes were greatest for depressive symptoms (OR 2.50, 95% CI 2.26–2.76), comorbidity count (3 + vs. 0 OR 1.68, 95% CI 1.47–1.91) and female sex (OR 1.46, 95% 1.37–1.55).

**Conclusion:**

Insomnia symptoms were not uncommon among comparatively younger individuals with typical OA joint symptoms and those with OA. This suggests that healthcare providers should address sleep-related issues in those consulting for joint pain, irrespective of diagnosis. Given their high prevalence, this also has implications for sleep-related issues at a population level.

## Introduction

Good quality sleep is an important factor for optimal cognitive functioning, memory consolidation, immune function and metabolic health [1–3]. However, insomnia symptoms, characterized as persistent difficulty with sleep onset or maintenance that may cause significant distress or impair daily functioning [4–7], are frequently reported in the population, with estimates ranging from 23–56% among adults [7,8]. Among Canadian adults, for example, between one-fourth to one-third report insomnia symptoms [9–11]. The continued experience of insufficient sleep can negatively affect both mental and physical health, as well as social participation and public and personal safety [1,2,12].

The association between pain and sleep has long been of interest as sleep disturbance is a highly prevalent complaint amongst those with chronic painful conditions [13–15], of which osteoarthritis (OA) is a prime example [16–20]. OA is one of the most common chronic diseases in the population and is associated with a number of negative health outcomes that reduce one’s quality of life and overall well-being [6,21–25]. The experience of OA-like joint symptoms without an OA diagnosis is also highly prevalent in the population. A recent population-based study noted that one-quarter of adults reported joint symptoms typical of OA but with no OA diagnosis [26]. Those with OA and those with joint symptoms but no OA diagnosis were found to be remarkably similar in sociodemographic characteristics, extent of comorbidities, and impact on health, except that the latter group was younger [26], which suggests that current estimates that only include those with diagnosed OA may underestimate the true impact of joint symptoms on sleep. In addition to OA, previous literature has also found female sex, comorbid chronic conditions and depressive symptoms to be strongly associated with insomnia symptoms [16,19,25,27,28].

Few studies have used population-based samples to specifically examine the association between OA and sleep problems, and these often rely exclusively on participants reporting doctor diagnosed OA. This potentially excludes individuals with milder disease who, though not having visited a healthcare provider and thus are lacking a diagnosis, are experiencing joint symptoms [23,26,29]. Using large, representative, population-based samples provides the opportunity to more completely represent the burden of OA and its effect on insomnia symptom prevalence and incidence, as these will include individuals with OA across the severity spectrum.

Many individuals often fail to discuss their arthritis or sleep problems with a healthcare professional [30–32] – a major deterrent is the anticipation of a negative or delegitimizing reaction from healthcare professionals about what may be perceived as less debilitating symptoms [33]. This is especially concerning for a rapidly aging population, which is also increasingly vulnerable to frailty, for example [34]. Recognizing the negative health consequences of these conditions and understanding the degree to which they may co-occur, including for those with OA-like joint symptoms but without a diagnosis, may spur providers to raise awareness and provide patient education about the availability of treatments. Furthermore, given their frequency in the population, this may highlight an opportunity to encourage the public to seek treatment for these conditions to reduce their impact.

This population-based study documents the frequency of insomnia symptoms among individuals with OA and among individuals with joint symptoms indicative of OA but not reporting an OA diagnosis. Additionally, it examines the likelihood of reporting insomnia symptoms by individuals with OA and those with joint symptoms but no OA, as compared to those with neither an OA diagnosis nor joint symptoms, adjusting for a range of factors associated with insomnia.

## Methods

### Data source

The study sample included individuals from the Canadian Longitudinal Study on Aging (CLSA) – Comprehensive Cohort. This is a national, longitudinal study that recruited a sample of Canadians who will be followed for up to 20 years. Participants in the CLSA were recruited by random digit dialing and from provincial health care registration databases by age, sex and urban or rural living classification. At the time of recruitment, all participants were aged 45–85, spoke English or French, and were cognitively unimpaired. The CLSA excludes those in the Canadian Armed Forces, living on Indigenous reserves or living in long-term care institutions. Full details of the CLSA study have been published elsewhere [35]. Baseline recruitment was completed from 2011–2015, and the current cross-sectional study uses data from the Comprehensive Cohort first follow-up (data collection completed from July 6, 2015 – January 9, 2019) questionnaire, in which sleep-related questions were introduced. Written informed consent was obtained from all CLSA participants prior to data collection. The present study received ethics approval from the University Health Network’s Research Ethics Board (#16–5883.8), and the data were first accessed for research purposes on May 1, 2024. The CLSA separately acquired ethics approval and this is renewed each year.

### Study design

**Fig 1:**
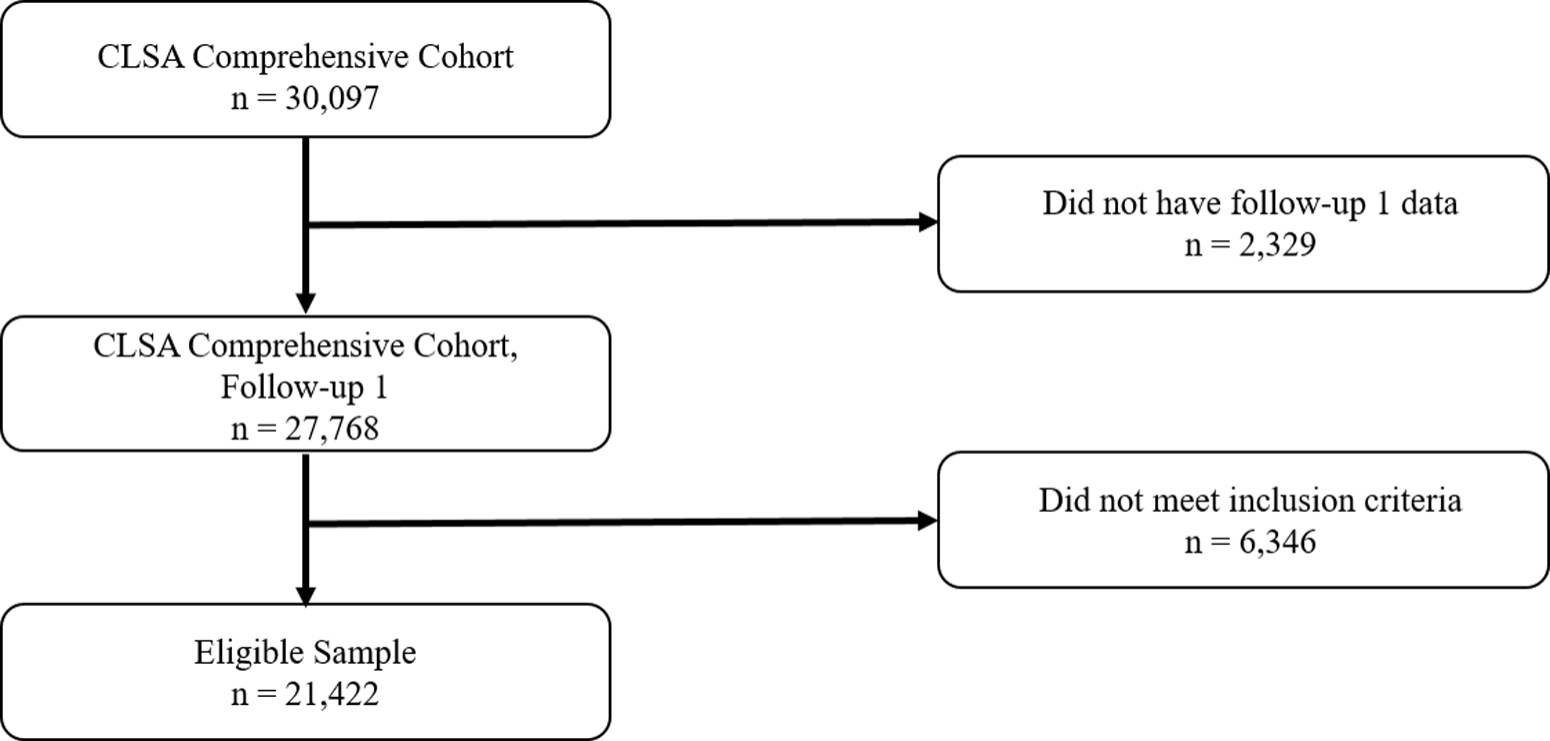
Participant selection.

The baseline survey was completed by 30,097 participants. Of these, 27,786 (92.3%) completed the follow-up 1 questionnaire. There were minimal differences in participant characteristics between those with and without follow-up 1 data. Those without were on average 4 years older, with lower household income and physical activity. No meaningful differences were found in, for example, sex distribution (female 50.9% vs 50.9%), body mass index (BMI normal 30.5% vs 28.0%, BMI obese 29.2% vs 31.6%), or OA status (OA 26.1% vs 27.8% and joint symptoms-no OA 20.6% vs 21.8%). 21,422 participants were eligible for the current study. Of these, 92.2% (19,749) had complete data (Fig 1). Missingness was largely due to missing household income data.

Participants were asked whether they had ever been told by a doctor that they had hand, hip or knee OA. In addition, all participants were asked a series of questions about joint symptoms typical of OA over the previous four weeks (pain most days in small joints near fingernails, pain most days in bases of thumbs, pain in groin or upper thigh, pain in groin or upper thigh walking down stairs or slopes, knee pain, pain in knee walking down stairs or slopes, or swelling in knee), irrespective of whether they reported an OA diagnosis. Using responses from these questions, individuals were placed into one of three mutually exclusive groups: those with an OA diagnosis; those with joint symptoms–no OA; and those with neither an OA diagnosis nor joint symptoms (controls). Individuals reporting any type of arthritis other than OA were excluded from the study.

Participants were also asked about how many times per week in the last month they required more than 30 minutes to fall asleep (sleep onset) and at which they wake up and have difficulty falling asleep again (sleep maintenance); each was recorded as never, less than once a week, 1–2 times a week, 3–5 times a week, or 6–7 times a week. Based on the DSM-5 criteria for insomnia [4] and available literature [36,37], responses to these questions were used to dichotomize individuals. Those who experienced difficulties with sleep onset or sleep maintenance at least three times a week were categorized as having insomnia symptoms, and those who experienced difficulties less than three times a week were categorized as having no insomnia symptoms.

### Covariates

Respondents were asked a number of questions pertaining to sociodemographic characteristics, health behaviors and health conditions. Gathered sociodemographic data included age, sex at birth (male/female), marital status (single, married or common-law, widowed or divorced or separated), and household income (CDN$ in ‘000s: <$50; $50 - <$100; $100 - <$150; >$150). Health behavior data included alcohol consumption (regular, occasional, non-drinker), smoking status (current, former, never), and level of physical activity. Physical activity was measured through the completion of a modified version of the Physical Activity Scale for the Elderly (PASE), which assigns a score based on frequency, duration, and intensity level of physical activity over the past week, where a higher score indicates a higher level of activity [38]. For analysis, PASE scores were categorized according to tertiles in the overall population distribution of scores (to reflect ‘lower’, ’moderate’, and ‘higher’ physical activity levels) to facilitate interpretability [39–41]. Body mass index (BMI (kg/m^2^)) was calculated from height and weight data, with individuals categorized as being normal (BMI 18–24.9), overweight (BMI 25–29.9) or obese (BMI 30+). Participants also reported whether they had been diagnosed with any chronic conditions, including cancer, cardiovascular, neurological, gastrointestinal, mental, and vision-related illnesses. For each participant, the total number of chronic conditions was calculated and categorized as 0, 1, 2, or 3+ conditions. Participants also reported whether they were experiencing depressive symptoms, irrespective of a depression diagnosis. Screening for depressive symptoms was completed with the Center for Epidemiologic Studies Short Depression Scale (CES-D-10), which contains questions about feelings of depression, loneliness, hopefulness for the future and restless sleep, where a score of 10 or more is categorized as having depressive symptoms [42]. For the purposes of this study, points associated with the question pertaining to restless sleep were removed from the calculated score to avoid confounding, and the remaining points were summed (0–27) [17].

### Statistical analysis

Descriptive statistics were generated for the study sample by OA status (i.e. OA diagnosis, joint symptoms-no OA, controls). All variables and an additional two auxiliary variables (level of education and self-rated health status) were used in a multiple imputation model in order to impute missing values. Logistic regression with fully conditional specification methods was employed for categorical variables. A total of ten imputed data sets were generated so that the number of imputations was a least equal to the percent of missing data for one or more variables. Logistic regression was employed to examine the association between OA status and our outcome of interest, insomnia symptoms (model outcome: yes/no), adjusting for covariates: sociodemographic factors, BMI, health behaviours and health conditions. Rubin’s rules were used in combining the estimates. Odds ratios (ORs) were calculated along with corresponding 95% confidence intervals (CIs). Additionally, as studies have reported sex differences in prevalence of OA, OA outcomes, and insomnia symptoms, the logistic regression model was re-estimated stratified by sex. Subsequently, a separate logistic regression model was estimated for those with OA (vs. controls) and for those with joint symptoms no-OA (vs. controls) to determine whether covariate effects differed between the groups. Required sample size for analysis was calculated based on the formula described by Vittinghoff et al. [43] for logistic regression analyses, using observed proportions of OA status and sleep problems and setting the minimum detectable odds ratio to 1.2, the false positive rate to 0.05, and statistical power to 0.80. The resulting required sample size was calculated to be 7,881, which was far exceeded by the available sample. Multicollinearity among independent variables was assessed by inspection of variance inflation factors (VIFs); estimates < 2.5 were interpreted as indicative of no problematic multicollinearity. All statistical analyses were completed using SAS 9.4.

## Results

### Descriptive statistics

**Fig 2:**
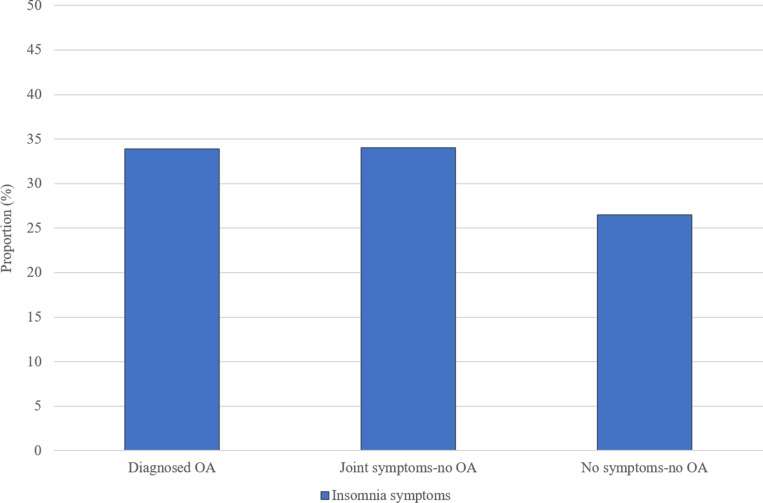
Prevalence of insomnia symptoms by OA status.

Table 1 displays the baseline characteristics of the study sample, overall and by OA status. The mean age of participants was 65.1 years and 49.3% were female. Diagnosed OA was reported by 29.1% of individuals, 17.2% reported having symptoms typical of OA but no OA diagnosis, and 53.7% reported having no OA or joint symptoms (controls). The same proportion of participants reported insomnia symptoms within each of the diagnosed OA and joint symptoms-no OA groups, 34.2%, compared to 26.7% among those with no symptoms-no OA (controls) (Fig 2). Those with OA and joint symptoms-no OA were also more likely to be female, obese, and have 3+ chronic conditions and depressive symptoms than controls. The diagnosed OA group had a higher mean age than the joint symptoms-no OA group and controls; these latter groups had a similar mean age.

**Table 1:** Distribution of baseline characteristics by OA status.

	Overall	OA Status			
	(N = 21,422)	Diagnosed OA (n = 6,230)	Joint symptoms-no OA (n = 3,691)	No symptoms-no OA (controls) (n = 11,501)	P-value^a^
	Percent (%)				
Insomnia symptoms	30.2	34.2	34.2	26.7	**< 0.001**
**Sociodemographic Characteristics**					
Age, mean (SD)	65.1 (10.0)	68.2 (9.3)	63.9 (9.9)	63.7 (10.0)	**< 0.001**
Female	49.3	60.9	48.8	43.1	**< 0.001**
Marital status					**< 0.001**
Single	9.1	8.3	9.8	9.4	
Married/common-law	69.2	64.7	70.0	71.4	
Widowed/Divorced/Separated	21.6	27.0	20.3	19.1	
Missing	< 0.1	<0.1	0	0.1	
Household income					**< 0.001**
<$50,000	23.5	28.7	23.8	20.6	
$50,000 – < $100,000	33.8	34.9	33.7	33.2	
$100,000 – < $150,000	19.3	16.4	19.0	21.0	
$150,000 +	17.7	12.8	17.9	20.2	
Missing	5.7	7.1	5.7	5.0	
**BMI**					**< 0.001**
Normal weight (BMI ≤ 24.9)	31.0	26.0	27.5	34.9	
Overweight (BMI 25.0–29.9)	34.0	37.9	40.1	41.1	
Obese (BMI ≥ 30)	27.6	34.5	31.5	22.7	
Missing	1.4	1.7	0.9	1.3	
**Health Behaviours**					
Alcohol consumption					**< 0.001**
Regular drinker	77.1	75.4	76.0	78.4	
Occasional drinker	11.3	12.2	11.7	10.8	
Non-drinker	11.5	12.3	12.3	10.8	
Missing	0.1	0.1	0.1	0.1	
Smoking status					**< 0.001**
Current	7.3	6.0	7.4	8.0	
Former	41.9	45.8	44.3	39.0	
Never	50.8	48.2	48.3	53.0	
Physical activity					
PASE, mean (SD)	138.9(73.6)	126.7 (68.6)	143.6 (74.9)	144.0(75.1)	**< 0.001**
PASE lower tertile (less active)	31.2	37.4	28.7	28.6	
PASE middle tertile	33.9	34.3	34.0	33.7	
PASE higher tertile (more active)	34.5	27.9	36.9	37.2	
Missing	0.5	0.5	0.4	0.5	
**Health Conditions**					
Chronic conditions					**< 0.001**
0	7.7	3.7	7.0	10.0	
1	14.5	8.8	13.5	17.9	
2	16.5	12.2	16.3	18.9	
3 +	61.1	75.2	62.9	52.9	
Missing	0.3	0.1	0.3	0.3	
Depressive symptoms (CES-D-10), yes	8.6	11.2	10.5	6.6	**< 0.001**
Missing	0.3	0.3	0.4	0.3	

^a^*P* values were calculated with independent two-sample t-tests for continuous variables and chi-square tests for categorical variables*. P* < 0.05 are bolded.

OA: osteoarthritis; BMI: body mass index; PASE: Physical Activity Score for the Elderly.

Table 2 displays the baseline characteristics of the study sample by insomnia symptoms status. Compared to individuals without insomnia symptoms, those with insomnia symptoms were more likely to report OA or joint symptoms-no OA, be female, have lower physical activity scores, and were nearly three times as likely to report depressive symptoms.

**Table 2:** Distribution of baseline characteristics by insomnia symptoms.

	Insomnia symptoms		
	Yes (n = 6,460)	No (n = 14,962)	*P* value ^a^
	Percent (%)		
**OA Status**			
Diagnosed OA	33.0	27.4	**< 0.001**
Joint symptoms-no OA	19.5	16.2	
No symptoms-no OA	47.5	56.4	
**Sociodemographic Characteristics**			
Age, mean (SD)	64.8 (10.0)	65.2(10.0)	**0.01**
Female	57.4	45.6	**< 0.001**
Marital status			
Single	9.6	8.9	**< 0.001**
Married/common-law	66.8	70.3	
Widowed/Divorced/Separated	23.7	20.8	
Missing	< 0.1	< 0.1	
Household income			
<$50,000	26.3	22.3	**< 0.001**
$50,000 – < $100,000	32.3	34.4	
$100,000 – < $150,000	18.9	19.5	
$150,000 +	16.2	18.3	
Missing	6.3	5.5	
**BMI**			
Normal weight (BMI ≤ 24.9)	31.4	30.9	**0.04**
Overweight (BMI 25.0–29.9)	38.6	40.6	
Obese (BMI ≥ 30)	28.3	27.3	
Missing	0.1	0.1	
**Health Behaviours**			
Alcohol consumption			
Regular drinker	75.4	77.8	**0.002**
Occasional drinker	12.4	10.9	
Non-drinker	12.2	11.2	
Missing	0.1	0.1	
Smoking status			
Current	8.6	6.8	**< 0.001**
Former	42.3	41.7	
Never	49.1	51.5	
Physical activity			
PASE, mean (SD)	134.1 (72.5)	141.0 (74.0)	**< 0.001**
PASE lower tertile (less active)	34.1	29.9	
PASE middle tertile	33.7	34.0	
PASE higher tertile (more active)	31.8	35.6	
Missing	0.4	0.5	
**Health Conditions**			
Chronic conditions			
0	5.4	8.7	**< 0.001**
1	11.5	15.8	
2	14.5	17.4	
3 +	68.3	55.0	
Missing	0.3	0.3	
Depressive symptoms (CES-D-10), yes	14.7	5.5	**< 0.001**
Missing	0.3	0.3	

^a^ *P* values were calculated with independent two-sample t-tests for continuous variables and chi-square tests for categorical variables*. P* < 0.05 are bolded.

OA: osteoarthritis; BMI: body mass index; PASE: Physical Activity Score for the Elderly.

### Regression analyses

Table 3 displays the results from the fully adjusted logistic regression analyses (all VIFs for the regression models were below the 2.5 threshold). Individuals with diagnosed OA had 25% increased odds (OR 1.25, 95% CI 1.17–1.35) of insomnia symptoms compared to controls. A very similar increased odds was observed for those with joint symptoms-no OA (OR 1.32, 95% CI 1.21–1.43). Other factors found to be significantly associated with the presence of insomnia symptoms were female sex, being a current smoker, being in the lowest physical activity tertile, having two or more chronic conditions, and reporting depressive symptoms. The magnitude of association was greatest for depressive symptoms (OR 2.50, 95% CI 2.26–2.76), which were similarly prevalent in the OA and joint symptoms-no OA groups, and more prevalent compared to controls.

**Table 3:** Multivariable logistic regression model results (outcome: insomnia symptoms yes/no).

	Odds ratio (95% CI)^a^
**Osteoarthritis status (vs. controls)**	
OA diagnosis	**1.25 (1.16-1.35)**
Joint symptoms-no OA	**1.32 (1.21-1.43)**
**Sociodemographic Characteristics**	
Age (per 10-year increase)	**0.99 (0.98-0.99)**
Female sex (vs. male)	**1.46 (1.37-1.55)**
Marital status (vs. married/common-law)	
Single	0.92 (0.82**-**1.03)
Widowed/Divorced/Separated	0.97 (0.89**-**1.05)
Income (vs. < $50,000)	
$50,000 - < $100,000	**0.90 (0.83-0.98)**
$100,000 - < $150,000	0.96 (0.87**-**1.07)
$150,000+	0.91 (0.82**-**1.02)
**Health Characteristics**	
BMI (vs. normal)	
Overweight (BMI 25.0–29.9)	0.98 (0.91**-**1.06)
Obese (BMI ≥ 30)	0.92 (0.85**-**1.00)
Physical activity (vs. PASE higher tertile)	
PASE lower tertile	**1.13 (1.04-1.23)**
PASE middle tertile	1.06 (0.98**-**1.14)
Alcohol consumption (vs. non-drinker)	
Regular drinker	1.00 (0.91**-**1.10)
Occasional drinker	1.01 (0.89**-**1.14)
Smoking status (vs. never)	
Current smoker	**1.22 (1.08-1.37)**
Former smoker	1.05 (0.99**-**1.12)
Chronic conditions (vs. none)	
1	1.16 (1.00**-**1.34)
2	**1.32 (1.15-1.53)**
3 +	**1.68 (1.47-1.91)**
Depressive symptoms (CES-D-10) (vs. none)	**2.50 (2.26-2.76)**

^a^ Significant odds ratios (95% CI does not contain 1.00) are bolded.

OA: osteoarthritis; BMI: body mass index; PASE: Physical Activity Score for the Elderly.

No difference in the effect of OA status on insomnia symptoms was found for males and females (OA vs. controls: male OR 1.20, 95% CI 1.08–1.34, female OR 1.31, 95% CI 1.19–1.44; joint symptoms-no OA vs. controls: males OR 1.29, 95% CI 1.15–1.45, female OR 1.35, 95% CI 1.21–1.52). Furthermore, the pattern of covariate effects on insomnia symptoms were found to be very similar for the OA and joint symptoms-no OA groups in the secondary logistic regression analyses (Table 4).

**Table 4:** Multivariable logistic regression model results (outcome: insomnia symptoms yes/no), stratified by OA status.

	Odds ratio (95% CI)^a^	
	Diagnosed OA	Joint symptoms-no OA
**OA Status (vs. controls)**	**1.24 (1.16-1.34)**	**1.32 (1.21-1.43)**
**Sociodemographic Characteristics**		
Age (per 10-year increase)	**0.99 (0.98-0.99)**	**0.99 (0.98-0.99)**
Female sex (vs. male)	**1.45 (1.35-1.56)**	**1.43 (1.33-1.55)**
Marital status (vs. married/common-law)		
Single	0.98 (0.86**-**1.10)	0.91 (0.80**-**1.04)
Widowed/Divorced/Separated	1.00 (0.91**-**1.10)	0.90 (0.81-1.00)
Income (vs. < $50,000)		
$50,000 - < $100,000	0.91 (0.83**-**1.00)	**0.86 (0.77-0.95)**
$100,000 - < $150,000	1.01 (0.90**-**1.13)	0.89 (0.79**-**1.02)
$150,000+	0.97 (0.86**-**1.10)	**0.86 (0.75-0.98)**
**Health Characteristics**		
BMI (vs. normal)		
Overweight (BMI 25.0–29.9)	1.00 (0.92**-**1.08)	0.96 (0.88**-**1.05)
Obese (BMI ≥ 30)	0.93 (0.85**-**1.02)	0.94 (0.85**-**1.04)
Physical activity (vs. PASE higher tertile)		
PASE lower tertile	**1.14 (1.04-1.25)**	**1.19 (1.08-1.31)**
PASE middle tertile	1.07 (0.99**-** 1.17)	**1.10 (1.01-1.21)**
Alcohol consumption (vs. non-drinker)		
Regular drinker	0.99 (0.89**-**1.10)	1.03 (0.91**-**1.15)
Occasional drinker	0.99 (0.87**-**1.14)	1.01 (0.87**-**1.17)
Smoking status (vs. never)		
Current smoker	**1.21 (1.07-1.38)**	**1.17 (1.04-1.36)**
Former smoker	1.06 (0.99**-**1.14)	1.07 (0.99**-**1.16)
Chronic conditions (vs. none)		
1	1.16 (0.99**-**1.36)	**1.20 (1.02-1.41)**
2	**1.34 (1.14-1.57)**	**1.41 (1.21-1.65)**
3 +	**1.73 (1.49-2.00)**	**1.71 (1.48-1.97)**
Depressive symptoms (CES-D-10) (vs. none)	**2.47 (2.21-2.77)**	**2.41 (2.13-2.74)**

^a^ Significant ORs (95% CI does not contain 1.00) are bolded.

OA: osteoarthritis; BMI: body mass index; PASE: Physical Activity Score for the Elderly.

## Discussion

From a large, population-based sample, we found insomnia symptoms to be frequent among individuals with an OA diagnosis, and to be equally frequent among those reporting joint symptoms but no OA diagnosis. This remained the case when controlling for potentially confounding factors such as sex, physical activity levels, and presence of chronic conditions and depressive symptoms. Unlike in much of the literature, we highlight that a large proportion of the population experiences joint symptoms typical of OA but reports no OA diagnosis, and that together, those with OA and those with joint symptoms-no OA represent a large segment of the population that may benefit from targeted approaches to improve sleep quality.

Adjusting for several relevant factors, the finding that those with an OA diagnosis have increased odds of experiencing insomnia symptoms is consistent with findings from others. Power et al. reported that those with arthritis had a 26% increased odds of reporting insomnia symptoms than those without arthritis [16]. Allen et al., studying individuals aged 45 and older from the Johnston County Osteoarthritis Project, found that individuals with symptomatic hip and knee OA had a 29% increased odds of reporting insomnia than those without symptomatic OA [27]. These reports are remarkably similar to findings from the current study, where those with OA has a 25% increased odds of reporting insomnia symptoms. In addition, among individuals with knee OA, Sasaki et al. reported that the prevalence of nocturnal knee pain increased with OA severity, and that severe knee OA was associated with increased sleep latency (the time it takes to transition from full wakefulness to sleep) (OR 1.18), and greater sleep disturbance (OR 1.19), relative to mild knee OA [18]. These sleep outcomes are comparable to the measures of difficulty with sleep onset and sleep maintenance, respectively, used in the current study to identify insomnia symptoms.

The prevalence of joint symptoms-no OA found in the current population-based study (17.2%) is also similar to that reported in the available, though limited, literature. A Canadian population-based study reported that 21% of adults had joint symptoms typical of OA but with no OA diagnosis [44], while a study of participants aged 45 + in a large urban U.S. city reported that 14% experienced chronic joint symptoms (pain, aching or stiffness in joints, not including the back or neck, persisting for three or more months) but had no previous arthritis diagnosis [45].

Few studies have compared any outcomes between individuals with typical OA joint symptoms but no OA diagnosis and those with an OA diagnosis, let alone specifically investigated associations with sleep problems. One study, based on data from the Observational Arthritis Study in Seniors (OASIS), of which participants were aged 65 and older, found that 14% and 58% of participants had difficulty with sleep onset or sleep maintenance three or more times a week, respectively, and this was the case for those who did and who did not meet the American College of Rheumatology criteria for knee OA but nevertheless reported similar levels of knee pain [19]. The current study, which includes a broader age range, provides additional evidence that insomnia symptoms are similarly as likely among those with joint symptoms but no OA diagnosis as they are among those with OA.

The prevalence estimates of insomnia symptoms reported in the current study are consistent with those of other arthritis-focused studies. Rothrauff et al. reported a prevalence of sleep trouble of 44% among those with OA and 24% among controls using NHANES data for respondents aged 60 and older [28]. Although similar to the estimates we report, 34.2% and 26.7%, respectively, sleep trouble in the Rothrauff et al. study was defined broadly as any complaint involving or concerning sleep, in contrast to the current study that focused specifically on insomnia-related symptoms. Power et al. reported that one-quarter of those with an arthritis diagnosis reported experiencing insomnia symptoms compared to 11% of those without arthritis [16]. The greater degree of difference between these groups relative to the current findings likely stems from the fact that their definition of arthritis included any arthritis type, whereas we focused on OA and excluded any other arthritis type. The evidence nevertheless points to insomnia symptoms as a common occurrence in rheumatic diseases. The prevalence of insomnia symptoms among those with joint symptoms-no OA (34.2%) was equal to that of those with diagnosed OA, but there is an absence of literature that provides this same comparison.

In addition to sharing the same prevalence of insomnia symptoms, we found those with joint symptoms-no OA and those with diagnosed OA to be remarkably similar in sociodemographic and health-related characteristics, with the exception that the former group was younger and proportionally less female. Yip et al. reported that individuals with typical OA joint symptoms but without a diagnosis were also similar to those with an OA diagnosis on the basis of number of co-occurring conditions and overall reports of pain and presence of functional limitations, despite the former group being younger [26]. Having adjusted for these factors, those with symptoms typical of OA but no diagnosis had highly similar increased odds of experiencing insomnia symptoms as those with a diagnosis, compared to those with neither. This suggests that OA population burden may be underestimated in some cases, as this group with typical OA joint symptoms is perhaps more likely to include some with undiagnosed OA, who potentially have OA earlier in the disease course or who do not have severe symptoms but nonetheless are experiencing associated negative health consequences, including poor sleep. They arguably also represent a group of individuals likely to be at higher risk of developing OA [46,47]. Thus, a population-level assessment of OA burden on sleep quality should likely not overlook this segment of the population with joint symptoms. Clinically, it also suggests that joint symptoms, particularly in younger adults, should not be ignored, as the impact of OA on a range of health outcomes appears to be similar across age groups [48] and the management and treatment approaches for joint symptoms are likely to overlap for those with and without an OA diagnosis.

Previous studies have documented that female sex and depressive symptoms are associated with poor sleep quality. Consistently, both a higher prevalence of insomnia symptoms and an adjusted increased likelihood of experiencing insomnia symptoms has been reported for women [5,11,16,25,28]. In line with these studies, we found that females were 1.5 times more likely to report insomnia symptoms than their male counterparts. Depression has also been reported to be independently and significantly associated with an increased likelihood of experiencing insomnia symptoms and sleep problems [16,27], and our results were consistent with these reports. Those with depressive symptoms were 2.5 times more likely to report insomnia symptoms than those without in our sample. Interestingly, we also found that the prevalence of depressive symptoms was similar between those with diagnosed OA and those with joint symptoms-no OA, and this prevalence was nearly twice that among controls. To our knowledge, this comparison has not been shown in previous studies. Associations between physical activity and sleep quality in those with arthritis have been inconsistent in the literature. While some cross-sectional studies have reported no association [11,16], a longitudinal study of participants from the Osteoarthritis Initiative (aged 45–79), using the PASE to capture physical activity (as done in the present study), reported that lower baseline physical activity scores (below the median) were more prevalent amongst those in the worsening restless sleep trajectory group relative to those in the ‘good’ trajectory group [49]. We found that those in the lowest tertile of PASE scores (lower physical activity) had elevated odds of experiencing insomnia symptoms compared to those in the highest tertile (higher physical activity). The joint symptoms-no OA group had the largest proportion of individuals in the lowest physical activity tertile.

Although not explored in this study, systemic inflammatory factors have been identified to have a role in the pathophysiology of OA and to be a consequence of OA, with inflammatory load appearing to be higher the greater the number of symptomatic joints in OA [50–54]. Systemic inflammation has also been identified as a possible contributor to sleep problems [55–57]. Thus, systemic factors, particularly low-grade systemic inflammation, may also provide a pathophysiological link between OA and sleep problems.

This study is based on a large population-based sample that, unlike many OA studies that are limited to older age groups (often 60 years and greater), included individuals across a broad age range, and is thus generalizable to a greater segment of the OA population. This study gave additional consideration to those in the population with joint symptoms typical of OA but without an OA diagnosis, which further broadens the generalizability as individuals with undiagnosed OA, or at high risk for OA, often are overlooked when assessing population-level burden. This also allowed for us to highlight that those with OA and joint symptoms-no OA are highly similar, except in age, and are at similarly increased odds of experiencing insomnia symptoms, and possibly other health outcomes.

A limitation of the study is that it relied on self-reported data, which can introduce misclassification or recall bias. For example, it is possible that individuals with OA may not have recalled an OA diagnosis or as of yet not received a diagnoses, and thus may have been included in the control group. This likelihood was reduced to a certain degree by our inclusion of individuals reporting joint symptoms but not an OA diagnosis. Even so, while it has been shown that for large-scale population surveillance purposes, self-reported OA is an acceptable method of measurement [58], the potential for these biases should not be overlooked. OA data available in the CLSA was specific to the hand, hip and knee. While these joints are the most frequently affected by OA, individuals with diagnosed OA or joint symptoms at other joint sites may have been misclassified into the control group. The consequence is that the magnitude of associations between insomnia and OA or joint symptoms derived from the regression analyses may be underestimates. In addition, while the CLSA joint symptoms questions were the same or similar to many OA-specific symptom measures used in the literature, we acknowledge that OA may not be the source of symptoms in all cases. The classification of insomnia symptoms was determined using CLSA questions that reflected DSM-5 criteria and the definitions of insomnia symptoms used in related studies. However, these were not part of a standardized clinical interview for the purpose of diagnosing insomnia, and further, information on “early-morning awakening” that is included in the DSM-5 [4] was not available and could not be considered within the study’s definition of insomnia symptoms. As noted above, sociodemographic and economic factors are important correlates of sleep problems. While we adjusted the regression analyses for household income and marital status, we recognize that this is nevertheless a narrow representation of SES, and the potential for residual confounding must be considered. Joint pain data was also limited in the CLSA. The inability to account for joint pain severity in the modelling means that residual confounding may remain; interpretations should be made accordingly. The CLSA cohort was limited to individuals at least 45 years of age and in addition did not include individuals living on Indigenous reserves. This limits the generalizability of our findings. We also acknowledge that individuals with arthritis other than OA were excluded in the current study. Thus, the findings may not generalize to those living with OA along with other types of arthritis. Finally, due to the cross-sectional nature of this study, temporality could not be assessed. Thus, we do not make causal, but rather associative inferences. In OA, the nature of the association between pain and poor sleep quality is complex, with some suggesting a possible bidirectional relationship, whereby joint pain can make it difficult to sleep, and poor sleep can subsequently decrease one’s ability to cope with pain and possibly heighten pain sensation [15,28,49,59–64]. We also recognize that factors such as physical activity and depression may be mediators in the pathway between OA and insomnia symptoms. However, given the cross-sectional nature of the data, this could not be formally tested.

This study, which includes a broad age range, provides evidence that those with joint symptoms typical of OA but not reporting an OA diagnosis and those with diagnosed OA were similarly more likely to report insomnia symptoms than controls, and were also highly similar in sociodemographic and health-related characteristics, except for age. This group of younger adults with joint pain may include individuals with undiagnosed OA or at higher risk of developing OA, which suggests that regardless of an OA diagnosis, information on joint pain burden and its effects in the population can help inform public health and healthcare system planning for strategies for targeted approaches to mitigate negative impacts on sleep and of poor sleep quality.

As noted, many individuals often neglect discussing their arthritis or sleep problems with their healthcare providers [30–33]. Recognizing that these conditions are concomitant, both for younger individuals with joint symptoms and older individuals vulnerable to increased frailty, provides an opportunity for provider initiated queries, and patient education about the availability of treatment. The literature highlights the primary importance of sleep in patient well-being, and our findings underscore that sleep should be assessed in OA. Optimal joint symptom management may be a key intervention for improved sleep quality and overall quality of life [65,66]. Given the frequency of OA and sleep problems in the population, the work also highlights an opportunity to encourage the public to seek treatment for these conditions to reduce their impact. Furthermore, on account of the complex interactions between sleep problems and OA, and depression, there is a need for more specific studies on the links between OA and sleep problems, and the outcomes of their treatments.
